# Face sensitivity: Effects of gender and orientation

**DOI:** 10.1192/j.eurpsy.2021.1957

**Published:** 2021-08-13

**Authors:** V. Romagnano, A. Sokolov, A. Fallgatter, M. Pavlova

**Affiliations:** Psychiatry And Psychotherapy, Eberhard Karls Universitaet Tuebingen, Tuebingen, Germany

**Keywords:** Non-verbal social cognition, Gender differences, Face sensitivity

## Abstract

**Introduction:**

Research on face tuning is of particular relevance during the Covid-19 pandemic leading to social isolation and anxiety, but also requiring social integrity. Face sensitivity represents an essential component of social competence. This ability is aberrant in most neuropsychiatric conditions. Studies in typically developing individuals enable to develop new tools for examination and better understanding non-verbal social cognition in neuropsychiatry.

**Objectives:**

Here we used a novel set of Face-n-Thing images to address the following issues: (i) whether the ability to seeing faces in non-face images (face pareidolia) is affected by gender; and (ii) whether it is altered with changing display orientation. The main advantage of Face-n-Thing images is that face tuning occurs without being explicitly fostered by familiar elements.

**Methods:**

A newly developed Face-n-Thing task, on which images were shown either with canonical upright orientation or inverted 180º in the image plane, was administered to healthy females and males. On each trial, they have to indicate whether they have a face impression.

**Results:**

Face impression was substantially impeded by display inversion in both males and females. With upright display orientation, no gender differences were found, whereas with inversion, Face-n-Thing images elicited face impression in females significantly more often.

**Conclusions:**

The findings open a way for examination of face sensitivity and underwriting brain networks in neuropsychiatric conditions, most of which are gender-specific. Display inversion represents a proper control for face tuning in neuroimaging studies. Gender differences should be taken into account when conceiving studies in neuropsychiatric populations.

**Disclosure:**

No significant relationships.

